# Experiences Accessing Health and Social Services during and after Natural Disasters among People Who Use Drugs in Houston, Texas

**DOI:** 10.3390/ijerph21091169

**Published:** 2024-09-03

**Authors:** Catherine E. Paquette, Tasia Danns, Margaret Bordeaux, Zaire Cullins, Lauren Brinkley-Rubinstein

**Affiliations:** Department of Population Health Sciences, Duke University School of Medicine, Durham, NC 27701, USAlauren.br@duke.edu (L.B.-R.)

**Keywords:** methadone, harm reduction, housing instability, social vulnerability, Texas, disasters, illicit drugs, poverty, natural disasters, cyclonic storms

## Abstract

People who use drugs (PWUD) disproportionately experience health-related and social vulnerabilities, which may affect service needs and access during and after natural disasters. We conducted qualitative interviews with *N* = 18 PWUD recruited via a syringe services program in Houston, Texas. We assessed their health and social service needs, as well as related service access experiences, during and after natural disasters using a combined inductive–deductive approach. Participants described a range of service-related needs related to illicit drug use, poverty, neighborhood disadvantage, acute and chronic health problems, and housing insecurity. They endorsed decreased access to medical and substance-related services and difficulty accessing disaster relief aid. Interviews highlighted the importance of mutual aid for sharing harm reduction supplies and meeting practical needs. Results suggest that some PWUD experience disproportionate vulnerability after natural disasters related to multiple marginalized identities that intersect with illicit drug use.

## 1. Introduction

As the frequency and severity of climate-related natural disasters increase in the context of global climate change, so too does the urgency of protecting populations who are most vulnerable. Climate-related disasters have increased five-fold over the past 50 years, causing 2.06 million deaths and USD 3.6 trillion in economic losses globally since 1970 [1]. Climate-related natural disasters include meteorological, climatological, and hydrological events such as storms, extreme temperatures (e.g., heat waves), drought, wildfires, and floods; such disasters have increased in probability due to anthropogenic climate change [1]. 

Natural disasters have a disproportionate negative impact on certain groups. Those who lack finances or a social safety net may have difficulty obtaining necessary resources and/or evacuating during disasters, as well as rebuilding and recovering afterward [2]. Groups that experience social and political marginalization are often made vulnerable due to the impact of stigma and discrimination in policy-making, resource allocation, and access [3]. Furthermore, those with physical and medical limitations are more destabilized due to the need for medical care (which is often disrupted during and after disasters), as well as greater difficulty mobilizing to find safety [2,4]. Thus, populations that have been identified as particularly susceptible to the effects of natural disasters include people who are low-income, homeless or unstably housed, racial/ethnic minorities, the elderly, people who are incarcerated or live in institutional settings, and individuals with substance use disorders [3,5,6]. 

People who use drugs (PWUD) may be at high risk of negative outcomes from natural disasters due to a constellation of factors related to health, housing, and socioeconomic status as well as drug use. PWUD are a vulnerable population “at risk of risks”; in other words, they experience compounding health risks that “generate exposure to other risks” [7]. Illicit drug use is associated with a range of acute and chronic health problems, including drug overdose, blood-borne infections, and chronic disease [8,9], care for which may be disrupted by natural disasters. PWUD also have high rates of housing insecurity and homelessness [10,11,12], which increases their risk of exposure to extreme temperatures and environmental hazards during these events. Furthermore, PWUD are more likely to reside in socially disadvantaged areas [13], which may contribute to significant environmental and economic vulnerability to disasters.

The challenges experienced by PWUD have significant implications for their support needs and ability to access services during and after natural disasters. For example, high rates of medical problems and homelessness suggest that PWUD may need healthcare and housing support in the wake of natural disasters. Despite this, there has been little research examining the service needs of PWUD and their related experiences after disasters. To address this gap in the literature, in this study we examined health and social service needs as well as related service access experiences during and after natural disasters among people who use drugs in Houston, Texas.

## 2. Study Setting

Houston, Texas, is a major metropolitan area located near the Gulf of Mexico that has notable vulnerability to climate-related disasters, including flooding. Houston’s particular susceptibility to flooding is exacerbated by its history of rapid urbanization, particularly urban development over environmentally sensitive areas such as wetlands [14,15]. The Federal Emergency Management Agency (FEMA) has declared 13 disasters in Harris County, where Houston is located, just since 2010; this includes three major floods, three hurricanes, and two ice storms [16]. Houston is located within the state of Texas, which has had the most flood-related deaths and damages of any U.S. state [14].

Previous research has found significant disparities in the impact of natural disasters on sub-groups in Houston and in Texas as a whole. For example, disadvantaged groups (including Hispanic immigrants) disproportionately reside in areas of Houston with higher flood risk [17,18], and areas of Texas with a high proportion of socially vulnerable residents are more likely to experience disaster-related casualties [19]. One study examined disparities in unmet service needs after Hurricane Harvey (2017) and reported that those with disabilities and those whose households lost jobs after Harvey had the lowest access to healthcare, but this study did not examine drug use among its vulnerability factors [20]. Indeed, very little published research has examined service needs or access among marginalized groups such as PWUD after natural disasters in the Houston region, representing a notable gap in the literature that this study aims to address.

## 3. Materials and Methods 

In July 2022, we recruited *N* = 18 PWUD via a syringe services program (SSP) in Houston, Texas, using a convenience sampling approach. Recruitment was prioritized in zip codes of Houston that had high rates of racial and ethnic polarization, operationalized as zip codes with the lowest index of concentration at the extremes (ICE) scores for race/ethnicity and income combined using data from the 2014–2019 American Community Survey [21]. Eligible participants were (1) aged > 18 at enrollment, (2) able to speak and understand English and to provide informed consent, and (3) met criteria for substance use disorder within the past six months (assessed via self-report). Potential participants unable to provide informed consent, including people with severe mental illness requiring immediate treatment or with mental illness limiting their ability to participate (e.g., psychosis), were excluded. Individuals receiving services from the Houston Harm Reduction Alliance SSP were informed about the study by SSP staff, and those who were interested were provided with study details and gave verbal consent to participate. Since all participants had a previous relationship with the SSP, any individuals who expressed or identified additional service needs during the screening, consent, or interview were referred back to the SSP for support and/or linkage to appropriate care. The study was granted a waiver for the requirement of written consent to ensure participant anonymity given the sensitive nature of the interview topics (e.g., illicit drug use) and minimal risk of participation apart from being identified as a study participant. 

Semi-structured qualitative interviews were conducted by a research assistant with expertise in qualitative methods in a variety of community settings (e.g., the SSP van, hotels, outdoors). They ranged in length from 16 to 68 min and lasted an average of 38 min. Topics in the interview guide focused on experiences with natural disasters, including experiences with (and observations about) accessing services during and after natural disasters, as well as participants’ substance use and perceptions of city services broadly. Prior to developing the interview guide, the research team conducted a literature review on the impact of natural disasters on the health and well-being of PWUD. Based on this literature review, we identified gaps in the research related to drug use and health, including PWUD service needs and access in the context of natural disasters. Questions in the interview guide were then developed with the goal of addressing these gaps in research. The interviewer also asked questions to assess participants’ demographics (race/ethnicity and age) and housing situation. Participants were compensated USD 50 for their time. Study protocols were approved by the Institutional Review Boards at the University of North Carolina and Duke University. 

Interviews were digitally recorded and transcribed verbatim. Transcripts were verified for accuracy by trained research assistants and deidentified. Interviews were coded using a combined inductive–deductive approach. The primary investigator (LBR) first created a “skeleton codebook” based on topics in the interview guide (inductive). The research team then conducted independent open coding on one full transcript, refining the codebook and adding additional codes based on emergent themes (deductive). The codebook was refined further during a second round of coding on an additional transcript. The final coding structure was applied to the full set of transcripts in Dedoose by two independent coders for each transcript, with quality checks to ensure high inter- and intra-coder reliability. 

For this analysis, we conducted a general inductive/thematic analysis [22] using NVivo Version 14 [23] to identify themes and participant experiences related to medical, health, and social services during and after natural disasters. We also examined codes related to resilience, problem-solving, and community to identify ways that participants coped with disasters and met their own needs as well as the needs of their communities during and after disasters. 

## 4. Results

### 4.1. Participant Characteristics

Of eighteen participants, ten self-identified as White or Caucasian, four as Black or African American, one as Hispanic, and three endorsed multiple racial or ethnic identities. Participants ranged in age from 29 to 61, with a mean age of 43. Participants endorsed use of multiple illicit drugs, including heroin and other opioids (*n* = 8), methamphetamine (*n* = 6), crack cocaine (*n* = 4), and marijuana (*n* = 3); participants commonly reported using multiple substances. When asked about their living situation, most participants (*n* = 12 of 16; housing status unavailable for *n* = 2 participants) described being homeless or unstably housed, including staying in temporary housing (e.g., motels) or on the streets.

### 4.2. Health Service Needs and Access

Participants described impaired access to a range of essential health services during and after natural disasters, including medical and SUD treatment, harm reduction services, and emergency response services. While some of the service needs described by participants were directly related to their drug use (e.g., harm reduction supplies), participants also described barriers to accessing care for other health conditions and noted how poverty and neighborhood-level disparities negatively affected their ability to meet these needs.

#### 4.2.1. Access to Medical Care 

Impaired access to medical care was a major theme, and interviews suggested that this was compounded by social disadvantage. Participants indicated that during natural disasters, medical services tended to be focused on addressing acute trauma and that this made it difficult to receive care for chronic conditions. One participant *[48 years old, Black]* highlighted how this particularly affected low-income individuals whose access to primary care may be limited due to finances:

*Participant: You know, I did email [about getting medical care] because I’m a diabetic*.

*Interviewer: Oh, so you’re diabetic and you needed medical care during [Hurricane] Ike?*

*Participant: I needed medical care. Hospital wasn’t hardly functioning, and if you wasn’t just… had a branch sticking out your hand and now got coldcocked by something that fell, head split open, or you just got money just to go there and have it taken care of… It take time, like a doctor, I can’t afford to go to the doctor.*

Similarly, another individual [51 years old, White] stated that they felt the city did not do enough to support people who need regular medical services for chronic conditions, resulting in many people being “cut off” from care during disasters. This participant also highlighted a perception of disparities in access to medical services based on neighborhood disadvantage:

*Let’s say [wealthy neighborhoods], they’ve got millions of dollars in the area. […] You guarantee you’re going to get better services out of that area because the amount of tax dollars, the amount that’s being put into it. If God forbid they flood, you’re going to have everybody and God come to there. You put it down here, let’s say [impoverished neighborhood] over here and they get flooded, well, there’s just not enough money.*

The perception of disparities in service access based on neighborhood was highlighted by multiple participants; neighborhood-level disparities were a significant factor when accessing medical and other health services for disaster support, and the neighborhoods where participants resided were cited as areas where disaster relief services, including health services, were disproportionately inaccessible.

Although participants mostly described decreased access to medical care, one individual described disaster relief workers who attempted to decrease barriers to accessing health services for PWUD by ensuring people knew that their services were not linked to law enforcement:

*When you did have like medical teams and units come through that would set up their little white little bone-looking things and going, “Hey, this is not to trap you to have you come here and we run your ID and find out you got warrants or searches, you got drugs on you. This here just to check you out physically”.*

This quote suggests a potential facilitator to accessing medical care during disasters (and thus represents an exception to the major theme of decreased care access), yet it is notable that the same participant reported extremely long wait times at these mobile medical units and indicated that overall, they found it very difficult to access post-disaster medical services.

#### 4.2.2. Access to SUD-Related Services

PWUD shared varied accounts about their experiences accessing SUD-related services, including medication for opioid use disorder (MOUD) and harm reduction supplies. Participants who had been taking MOUD during disasters highlighted the importance of “take-home” doses of methadone during disasters (whereas daily visits to a methadone treatment clinic are typically required) and how access to take-home doses before disasters was inconsistent. One participant [35 years old, White, primary drug heroin] praised a methadone clinic for proactively providing a week’s worth of medication in anticipation of an ice storm. However, when participants did not have access to take-home doses, it resulted in significant withdrawal symptoms as well as anxiety and stress. Another individual [61 years old, White] shared about their inability to obtain methadone after Hurricane Harvey:

*However, Harvey, which… I don’t know if you remember. It flooded the whole city up. you couldn’t get your medication [for opioid use disorder]. […] It was a majorly disaster. Finally, the doctor at the [medical clinic] opened up a clinic at the [public location], and he was giving methadone out to people, but other than that, you were screwed if you didn’t have anything. […] People were getting sick and everything. […] Of course, you panic. You’re scared. You’re not sure what’s going to happen.*

Sterile injection supplies were identified as among the most important services to provide to PWUD after disasters, yet participants described difficulty obtaining harm reduction supplies from both the SSPs and pharmacies. One individual [35 years old, White] noted that the mobile harm reduction services that usually delivered naloxone were not able to reach PWUD during disasters, and this was described as particularly problematic because this individual believed there was an increased likelihood of overdose due to disaster-related stress:

*Because you’re doing more drugs at that time [of the disaster]. It will probably be your stress levels going to play a big part in it, too. […] some people might start doing more heroin, they can OD [overdose]. Because they’re doing too much, because they’re sad. You know what I’m saying? So they want to numb the pain more. And they can do a little bit too much. And then, there’s not no Narcan, because- because it’s hard to get it.*

Notably, another participant reported giving free syringes and naloxone to other people who use drugs when the SSP could not reach participants after storms; the participant described encouraging community members to carry naloxone even if they themselves did not need it, in order to be prepared to help others. 

### 4.3. Practical Needs and Social Service Access

#### 4.3.1. Importance of Housing and Shelter

Regarding social service needs during and after natural disasters, participants frequently highlighted the importance of housing and homelessness services. Multiple individuals described specific vulnerability experienced by those who were unhoused during disasters and shared personal experiences navigating these challenges. For example, when asked what kinds of services are most needed after disasters, a participant who was staying at a motel [31 years old, White] cited homeless shelters as among the most important and noted that this was particularly vital in the neighborhood where they resided due to high rates of homelessness. Yet structural and policy-level barriers sometimes prevent people in these neighborhoods from accessing shelter during disasters. For example, it was noted that homeless individuals were subject to restrictive policies such as strict check-in times or restrictions on bringing family members or pets into shelters. 

#### 4.3.2. Mutual Aid

A clear theme of mutual aid emerged regarding the ways in which participants met practical needs for themselves and their unhoused neighbors in the context of disasters, despite that mutual aid was not a topic originally addressed in the interview guide. Similar to the individual who reported giving out harm reduction supplies, multiple participants described a sense of their communities “coming together” to help each other with needs such as food, housing, and hygiene. For example, a participant who reported being homeless [39 years old, Hispanic] indicated that people in their neighborhood had provided food and water for those who were unhoused after natural disasters. Another individual who rented a hotel room [35 years old, multiracial] shared a story of allowing neighbors who were unhoused to take showers in their hotel room after one disaster. A third participant [52 years old, Black] shared a story of how their neighbors had offered to buy them food and supplies before a hurricane, when the participant had been more concerned with obtaining drugs and had not been prioritizing other basic needs. Aid was sometimes even extended to individuals from outside their communities; for example, one individual [42 years old, multiracial] reported that after Hurricane Katrina, neighbors helped provide shelter for strangers who had evacuated from Louisiana. The participant stated:

*Texas people have a hospitality. […] And when stuff gets going wrong like that, like a disaster, we can open up our hearts and our homes to people and take people in*.

#### 4.3.3. Post-Disaster Financial Support 

Despite the social and financial vulnerability experienced by many participants, most reported that they had never applied for any forms of government assistance after a natural disaster. Participants cited a perception of inaccessibility and structural barriers to the process of applying for social services and disaster-related aid. A few noted that they had tried applying for various types of government aid outside of disaster situations but had been unsuccessful. Examples included attempts to apply for unemployment benefits, food stamps, free cell phones, and child welfare assistance. They described long application wait times and difficult-to-navigate bureaucracy, which dissuaded some from trying to apply for disaster-related assistance. One participant who reported having a college education [51 years old, White] described how difficult it was to identify the right phone number to call when applying for aid:

*Applying for [disaster-related government aid], I thought it was just, oh man, the city’s too big. […] It’s too big. When you’re trying to apply for services, okay, yeah, I mean, you’re trying to get either aid, whether it be money, food, shelter, stuff like that. Well, either the phone lines they’re overwhelmed so they shut down. You can’t get ahold of somebody. You don’t know who to call. […] No. I mean, you look up right now, even on the internet, I mean, you look up Houston city services. Okay. I tried to look up child welfare. There must be 40, 50 numbers in that and it’s just- […] Literally 50 numbers*.

Only two individuals reported having started the application process for aid after a disaster. A participant who was homeless [40 years old, White] indicated that their application process was stymied because they had no internet access. Another homeless participant [35 years old, White] endorsed having applied for relief funds from FEMA but stated that the application process was long and that their contact information changed due to getting a new phone number after starting the application, which may have prevented the application from moving forward. Ultimately, neither was successful in obtaining disaster-related assistance.

## 5. Discussion

In this study, we qualitatively assessed experiences with health and social service needs and access during and after natural disasters among people who use drugs in Houston, Texas. Our sample consisted primarily of people who were homeless or unstably housed, used and injected criminalized drugs, and who resided in economically disadvantaged areas with high rates of racial and ethnic polarization. Participants described some service needs directly related to their drug use (e.g., MOUD and harm reduction supplies), but also endorsed a range of needs related to other aspects of social vulnerability, including homeless shelters and affordable care for chronic medical conditions. Numerous structural barriers to service access were cited, including neighborhood-level disparities in service provision, policy barriers to homeless shelter access, financial barriers to medical care, and logistical barriers to receiving financial assistance. Results highlight how PWUD may experience multiple aspects of social and economic marginalization that can increase vulnerability during natural disasters.

Natural disasters are a category of “Big Events” (also called “complex emergencies”; [24]) that can cause significant disruption in social systems and service infrastructure; previous research and theory suggest that these sequelae of Big Events can lead to serious adverse consequences for PWUD after natural disasters, including HIV outbreaks [25,26]. Consistent with this body of literature, participants in our study described impaired access to a range of essential medical and harm reduction services after natural disasters, which may have serious health implications. For example, previous research has shown that PWUD may engage in riskier drug injection practices due to a lack of harm reduction supplies (e.g., sterile syringes) after disasters [27]. One participant in our study was particularly concerned about the inaccessibility of naloxone after hurricanes because they believed that the risk of overdose may be heightened as people use more drugs to cope with stress. Indeed, a previous study has also identified a higher overdose incidence after Hurricane Maria [28], although there is mixed evidence on the extent to which drug use increases after disasters [28,29]. Our study builds on previous research demonstrating that people who use MOUD experience treatment interruptions after major storms. Previous studies have found that this can lead to obtaining medications or illicit drugs from sources other than their prescriber or trusted sellers [27,30], which may also increase overdose risk via exposure to street drugs and medications of varied potency. Thus, our study adds to the small body of literature demonstrating the importance of including specific provisions for MOUD and harm reduction services in disaster preparedness plans. For example, disaster relief plans could specifically include guidance for service provision to PWUD and present multiple options for naloxone distribution, other harm reduction, social and health services, and treatment. This should include provisions for take-home doses of methadone, which were highlighted as a facilitator of treatment access by one participant in our study and have been identified in previous research as an important service delivery adaptation for PWUD during Big Events [31]. Our study also suggests that efforts to unlink mobile medical services from law enforcement may increase accessibility for people who experience criminal legal vulnerability, including PWUD.

Notably, PWUD health and service needs intersect with socioeconomic disadvantage and geography. Participants in our study noted logistical and financial barriers to accessing care for chronic medical conditions and described neighborhood-level disparities in the provision of mobile health services after disasters. Previous research has found that individuals in communities with greater socioeconomic vulnerability disproportionately rely on local service infrastructure for medical care after disasters, which is likely to be significantly disrupted, whereas those who are wealthier may be more likely to evacuate and seek care in less affected areas [32]. Together, this underscores the importance of providing low-barrier health services in under-resourced areas after disasters. Participants in our study also described structural barriers to accessing disaster-related financial assistance, which compounded with their own lack of resources (e.g., cell phone and internet access) to prevent participants from benefiting from government assistance. Thus, despite that under-resourced areas of Houston tend to have greater susceptibility to natural disasters [17,18], these communities may also be disproportionately unable to access the government programs meant to help them after disasters.

The wide range of service needs and related access challenges endorsed by participants in this study underscores the multifactorial vulnerabilities faced by some PWUD. This highlights the importance of applying a “vulnerable population” intervention approach in the context of natural disasters, which is an approach that aims to decrease health inequalities by addressing the social and environmental conditions that contribute to unequal risks experienced by vulnerable groups [7]. Intersectoral collaboration has been identified as a key aspect of the vulnerable population approach [7] and may be especially vital in natural disaster response. Intersectoral approaches for natural disasters emphasize collaboration between diverse actors, including mental health workers, faith-based groups, emergency personnel, government, and public health workers [33]. Importantly, these approaches are well-suited to address both immediate needs and impacts (e.g., safety, food, and shelter) as well as longer-term impacts of disasters on vulnerable groups (e.g., substance use, mental health, poverty). When developing intersectoral approaches for vulnerable populations such as PWUD, it is important to consider training and technical assistance for service providers and other actors; for example, our study suggests that medical workers who recognize PWUDs’ potential fears about criminalization can take steps to reduce barriers to accessing medical care.

Finally, our study also highlighted the ways in which PWUD and people in disadvantaged areas support each other, which may be particularly important when the services and systems intended to offer support fail to meet the needs of these communities. Previous research has highlighted the importance of social connectedness for disaster-related resiliency in predominantly Black neighborhoods of Houston, where residents experience disproportionate social vulnerability to disasters [34]. Our study suggests that PWUD may engage in similar mutual aid efforts, including distributing harm reduction supplies to each other when harm reduction programs are interrupted by natural disasters. This is consistent with a body of literature documenting a high prevalence of informal mutual aid and “intravention” practices among people who inject drugs, including providing condoms, naloxone, and sterile injection supplies to one another (e.g., [35,36,37,38]). When developing plans to support PWUD after disasters, people with lived and living experience of drug use must be centered in planning processes, as they both know the needs of their communities and may be best situated to reach other PWUD with necessary resources and support. Disaster preparedness plans could consider intentionally engaging PWUD via peer support models to help extend the reach of disaster relief efforts to these often difficult-to-reach populations. This is also consistent with the vulnerable population approach to intervention, which prioritizes participation from directly impacted groups [7]. Future research should also further evaluate the ways in which PWUD and their communities support one another during disasters to develop a more nuanced understanding of how these interactions unfold and potential ways to build on these informal support structures during Big Events in the future.

### Limitations

Our study has limitations. We recruited a convenience sample of PWUD through an SSP, and our sample may not be representative of all PWUD in Houston or elsewhere. Some of our participants had limited experience accessing services after disasters, and we did not collect detailed data on participants’ experiences with specific climate-related disasters, so we were not able to assess how these experiences may have changed over time or how they were related to specific disaster preparedness/response policy changes in Houston. Additionally, the interviews were relatively brief (lasting less than 40 min on average), and participants were interviewed only once each; these aspects of the methodology may limit the depth of the data collected and the interviewer’s ability to form trusting relationships with participants.

## 6. Conclusions

Some PWUD experience disproportionate vulnerability after natural disasters, which is compounded by neighborhood disadvantage, homelessness, chronic health problems, and the need for consistent access to medications for opioid use disorder. Disaster relief plans must address multiple aspects of structural disadvantage to ensure the health and well-being of vulnerable groups, such as PWUD.

## Data Availability

Due to the sensitive nature of the qualitative interviews, data included in this study will not be made publicly available.
